# Integrative diagnosis of psychiatric conditions using ChatGPT and fMRI data

**DOI:** 10.1186/s12888-025-06586-w

**Published:** 2025-02-19

**Authors:** Runda Li

**Affiliations:** Vanderbilt University, 2301 Vanderbilt Place, Nashville, 37235 TN USA

**Keywords:** Psychiatric diagnosis, Natural language processing, ChatGPT, FMRI, Neural network, Machine learning, Mental health

## Abstract

**Background:**

Traditional diagnostic methods for psychiatric disorders often rely on subjective assessments, leading to inconsistent diagnoses. Integrating advanced natural language processing (NLP) techniques with neuroimaging data may improve diagnostic accuracy.

**Methods:**

We propose a novel approach that uses ChatGPT to conduct interactive patient interviews, capturing nuanced emotional and psychological data. By analyzing these dialogues using NLP, we generate a comprehensive feature matrix. This matrix, combined with 4D fMRI data, is input into a neural network to predict psychiatric diagnoses. We conducted comparative analysis with survey-based and app-based methods, providing detailed statistical validation.

**Results:**

Our model achieved an accuracy of 85.7%, significantly outperforming traditional methods. Statistical analysis confirmed the superiority of the ChatGPT-based approach in capturing nuanced patient information, with *p*-values indicating significant improvements over baseline models.

**Conclusions:**

Integrating NLP-driven patient interactions with fMRI data offers a promising approach to psychiatric diagnosis, enhancing precision and reliability. This method could advance clinical practice by providing a more objective and comprehensive diagnostic tool, although more research is needed to generalize these findings.

## Introduction

### Background and motivation

Psychiatric disorders represent a significant burden on global health, affecting millions of individuals and imposing substantial economic costs. Traditional diagnostic methods often rely heavily on clinician judgment and patient self-reporting, which can be subjective and inconsistent. Other methods, such as app-based or paper-based surveys, often fail to capture the depth and nuance of patient experiences due to their rigid and non-interactive nature. These methods can result in incomplete or superficial data, hindering accurate diagnosis.

Despite the promise of purely quantitative approaches, current psychiatric assessment frameworks still face challenges in integrating multiple data types—including text-based, behavioral, and imaging data—into a comprehensive diagnostic procedure. A *mixed-methods strategy*, which balances both quantitative and qualitative approaches, could mitigate some of these issues but introduces the complexity of handling heterogeneous datasets. Consequently, there is a need for novel methods capable of unifying these diverse data sources into a single, robust framework.

Moreover, existing diagnostic tools often fail to capture subtle cues such as changes in tone, pauses, or expressive language, all of which can be crucial for identifying underlying psychiatric conditions. This gap highlights the importance of enhancing the objectivity, consistency, and detail of patient assessment data, especially for high-stakes clinical decisions.

### Technological integration

Recent advancements in artificial intelligence (AI) and neuroimaging present an opportunity to enhance the diagnostic process. NLP techniques, particularly those involving conversational agents like ChatGPT, can systematically extract clinically relevant information from patient dialogues. Concurrently, functional magnetic resonance imaging (fMRI) provides detailed insights into brain activity patterns associated with psychiatric conditions. Integrating these modalities could lead to more accurate, objective, and timely diagnoses.

AI-driven image analysis can enhance the interpretability and diagnostic utility of neuroimaging data. For example, convolutional neural networks (CNNs) have been successfully applied to fMRI data to detect abnormalities and predict disease states [1]. The integration of AI and neuroimaging is not just limited to diagnosis but also extends to prognosis and treatment planning, offering a comprehensive approach to mental health care [2]. Recent advancements highlight the role of AI in neuroimaging, showing potential to revolutionize psychiatric diagnostics [3]. In parallel, advanced NLP methods can pick up subtle linguistic cues in patient narratives that conventional surveys cannot [4]. This synergy across modalities could address the primary shortcomings in current psychiatric evaluation strategies: lack of depth, subjective variability, and limited scalability.

### Research objectives

This study aims to explore the feasibility and efficacy of using ChatGPT to enhance patient interviews by capturing detailed linguistic and emotional cues, which are then quantitatively analyzed alongside fMRI data for psychiatric diagnosis.

We focus on addressing current gaps by discussing how the quantitative integration of fMRI data and qualitative conversational data in a single predictive framework can enhance diagnostic outcomes. The study highlights that advanced NLP captures subtle nuances in patient language beyond the scope of traditional app-based or survey-based assessments, and demonstrates how such improved data inputs translate into meaningful diagnostic gains that could impact real-world clinical outcomes.

### Structure of the paper

This paper is structured as follows: the following section reviews related work in the fields of NLP and neuroimaging for psychiatric diagnosis. After that, we detail our methodology, including data collection, feature matrix construction, and neural network architecture. We then present our results, including model performance and an expanded comparative analysis against existing diagnostic approaches. This is followed by a discussion of the clinical implications, limitations, and potential future directions for research. We conclude with a summary of our findings and their significance for psychiatric diagnostics.

## Related work

### NLP in clinical contexts

NLP has been increasingly applied in healthcare, with significant advancements in clinical text analysis and patient interaction systems. Recent studies have demonstrated the effectiveness of NLP in extracting symptoms, tracking disease progression, and supporting diagnosis. Shickel et al. (2018) highlighted the potential of deep learning approaches in processing electronic health records to identify patterns indicative of various medical conditions [5]. Esteva et al. (2019) utilized NLP to analyze patient-doctor conversations, providing insights into patient symptoms and concerns that might not be captured through standard clinical questionnaires [6]. Additionally, work by Savova et al. (2010) demonstrated the utility of NLP in extracting phenotypic information from clinical narratives, aiding in the development of large-scale phenotyping algorithms [7]. More recently, Johnson et al. (2020) showed how NLP can be used to identify suicidal ideation from social media posts, illustrating the broader applicability of these techniques beyond traditional clinical settings [8]. Advances in AI and NLP continue to expand the capabilities of psychiatric diagnostics, integrating conversational agents and machine learning for better outcomes [9].

### Neuroimaging for psychiatric diagnosis

Neuroimaging, particularly fMRI, has been pivotal in understanding the neural underpinnings of psychiatric disorders. Research has shown that fMRI can identify distinct brain activity patterns associated with conditions such as depression, anxiety, and schizophrenia. Abé et al. (2018) demonstrated the potential of combining fMRI data with machine learning algorithms to improve diagnostic accuracy for psychiatric disorders [10]. Drysdale et al. (2017) provided a groundbreaking approach to identifying biomarkers for depression subtypes using fMRI data, significantly advancing personalized medicine in psychiatry [11]. Wolfers et al. (2015) integrated multimodal imaging techniques to enhance the understanding of complex psychiatric conditions, highlighting the importance of comprehensive data integration [2]. Recent studies continue to underscore the promise of neuroimaging combined with AI for precision psychiatry [12, 13].

### Integrative approaches

Despite the advancements in NLP and neuroimaging, the integration of these modalities for psychiatric diagnosis remains relatively unexplored. Previous attempts have been limited to combining text-based clinical data with neuroimaging features in separate analyses. Recent studies, however, suggest the potential benefits of a more integrated approach. Schnyer et al. (2019) explored the integration of neuroimaging data with electronic health records, showing promise in improving diagnostic precision for mental health conditions [14]. Koutsouleris et al. (2018) demonstrated that combining neuroimaging data with clinical assessments can enhance the predictive accuracy of psychosis onset [15]. The integration of AI and neuroimaging is poised to transform psychiatric diagnostics, as evidenced by recent advancements [16, 17].

Many integrative studies still face difficulties in seamlessly combining qualitative text-based insights and high-dimensional neuroimaging features. NLP-driven analyses risk missing key contextual or emotional elements if they rely solely on structured questionnaires or static text, whereas neuroimaging alone may not capture the complex affective and social dimensions of mental health. Multimodal approaches that combine the strengths of NLP and fMRI could address these shortcomings, leading to more reliable and interpretable diagnoses.

## Methodology

### Experimental design

**Fig. 1:**
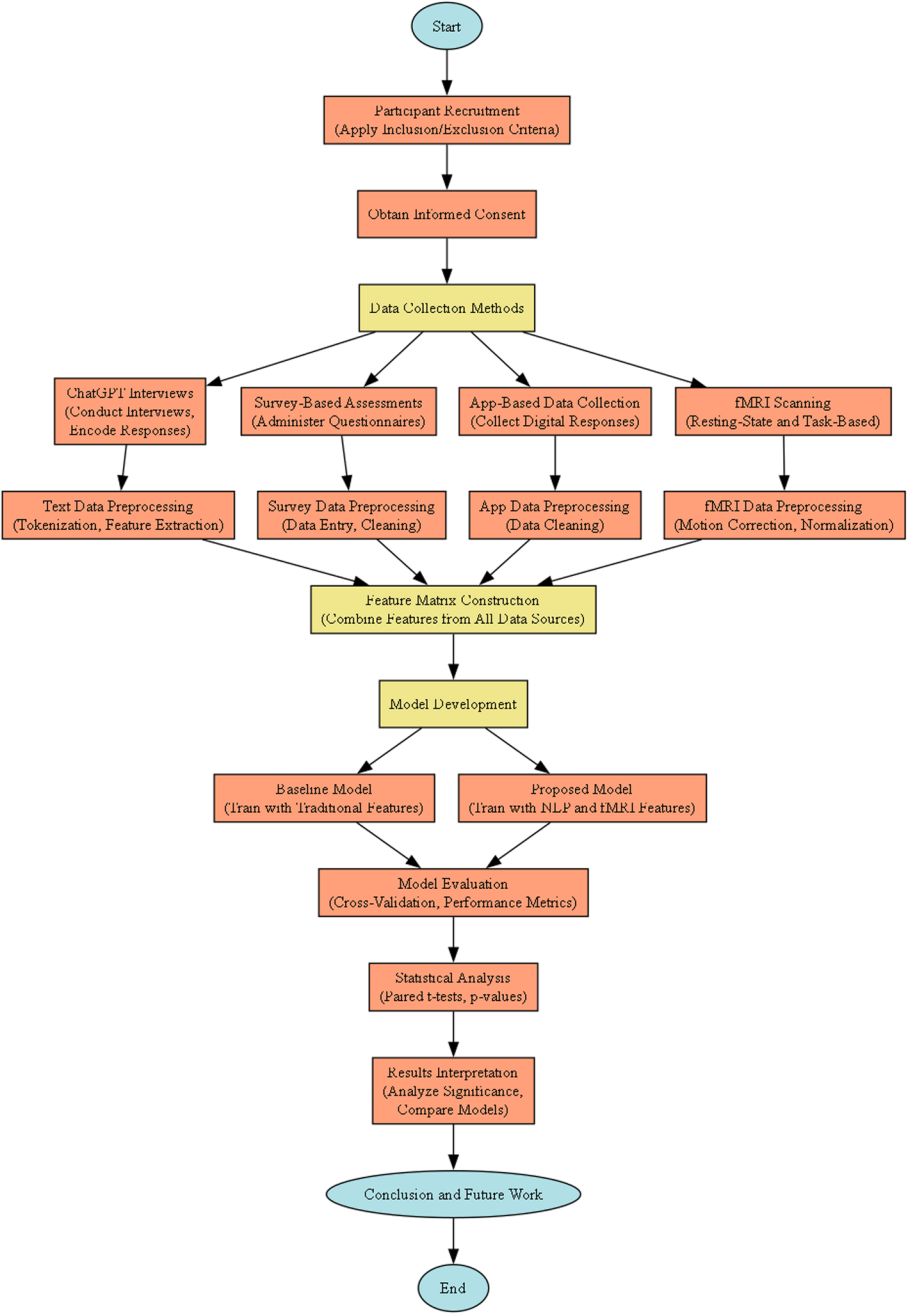
Experimental design workflow

Figure 1 presents the general workflow of our experimental design. The data collection stage involves patient interactions with ChatGPT and fMRI data acquisition. Processed results from both text and imaging pipelines are then transformed into feature matrices, which are subsequently used to train a neural network for evaluation. The final stage assesses model performance and carries out statistical validation.

### Data collection

In this study, ChatGPT plays a crucial role in collecting detailed and standardized patient information through dynamic and interactive interviews. Unlike traditional methods such as app-based or paper-based surveys, which provide structured but rigid data, ChatGPT’s conversational approach adapts to patient responses in real time. This adaptability allows the AI to probe deeper based on initial answers, uncovering latent symptoms and concerns that standardized questionnaires may overlook.

A mixed-method approach combines quantitative data (including numerical encodings such as Likert-scale responses) with qualitative insights captured from the free-form patient dialogues. This strategy blends the depth of qualitative investigations with the objectivity of quantitative assessments, offering a more holistic view of the patient’s mental state [18].

By adhering to a consistent interviewing script while allowing for natural language variations, ChatGPT ensures that all patients are engaged in the same core areas of inquiry. This uniformity includes questions about mood, anxiety levels, sleep patterns, and medical history. Because all patients experience the same set of guided topics, interviewer bias is minimized, which is particularly important given the subjectivity of psychiatric diagnosis.

ChatGPT also provides a level of comfort and engagement for patients who may feel more at ease disclosing sensitive information to an AI interviewer. Studies, such as Lucas et al. (2014), indicate that reduced fear of judgment can lead to greater disclosure [19]. This openness can result in richer data, which more thoroughly reflects the patient’s condition.

In addition to obtaining responses, ChatGPT uses advanced NLP techniques to extract and quantify nuanced details from the dialogue. Linguistic markers correlated with psychiatric disorders, such as significant shifts in sentiment or changes in pronoun use, are automatically captured [20]. Traditional surveys often overlook these subtle linguistic signals, thereby limiting their diagnostic potential.

Once the data is collected, the conversational content is parsed and transformed into a numerical feature matrix. Responses are tokenized, lemmatized, and classified into standardized categories such as mood descriptors or behavioral indicators. This ensures consistency and comparability across different patients and sessions, allowing for the integration of dialogue-based data with neuroimaging measures.

By merging both qualitative and quantitative information into a single pipeline, the method mitigates the pitfalls of purely narrative or purely numerical approaches and produces a robust, richly textured dataset. The resulting data is then ready for subsequent model training and predictive analysis.

#### fMRI data acquisition

fMRI scans were conducted to capture brain activity patterns associated with psychiatric disorders. Resting-state fMRI identified baseline neural activity while participants lay still with eyes closed, focusing on default mode network and other intrinsic connectivity networks. Task-based fMRI helped probe specific cognitive and affective processes. In an emotional recognition task, participants viewed images of faces expressing different emotions (e.g., happiness, sadness, fear, anger) and identified them, thereby activating the amygdala and prefrontal cortex. A memory recall task required participants to memorize a list of words or images and then recall them after a delay, targeting the hippocampus and surrounding medial temporal lobe structures. An attention and inhibition task, such as the Stroop test, prompted participants to name ink colors while ignoring the lexical meaning of the words, activating the anterior cingulate cortex and dorsolateral prefrontal cortex.

Raw fMRI data were preprocessed to correct for motion, align images to a common template through spatial normalization, and reduce low-frequency noise via temporal filtering. By standardizing these procedures, data quality was improved and made consistent across all study participants, enabling subsequent feature extraction focused on regional brain activation and connectivity patterns.

### Feature matrix construction

#### Baseline model features

A baseline model was also developed, relying on traditional clinical features rather than NLP-derived data. The baseline features included demographic information such as age and gender. They also encompassed clinical assessments, including questionnaire-based scores (PHQ-9 for depression and GAD-7 for anxiety) and symptom severity ratings, as well as basic self-reported changes in sleep, appetite, and energy. These baseline data points were assembled into a numerical matrix analogous to the main model but did not include the richer NLP-derived features from patient dialogues.

#### Feature extraction from patient dialogues

Dialogue-based features were extracted using NLP techniques that standardized and encoded the patient responses. Tokenization, lemmatization, and category mappings were used to ensure uniform data treatment. Mood descriptions and sleep patterns were converted into behavioral indicators, and linguistic cues such as hesitation, negation, or sentiment shifts were also encoded. This uniform approach minimized variability across patients and allowed direct comparisons across a wide variety of qualitative inputs.

#### Preprocessing fMRI data

Preprocessing of fMRI data included motion correction to compensate for participant movements, spatial normalization to map images onto a common brain template, and temporal filtering to remove low-frequency noise. These steps culminated in consistent volumetric maps of brain activity across patients. The finalized fMRI data was then used to extract features such as regional activation levels and brain connectivity patterns, formatted for subsequent integration with the patient dialogue features.

#### Patient selection criteria

Participants in the study were required to have a confirmed diagnosis of a single psychiatric disorder, be at least 18 years old, and be proficient in Mandarin Chinese. Only those capable of informed consent and willing to undergo both ChatGPT interviews and fMRI scanning were included. Anyone with severe neurological disorders, acute psychiatric symptoms requiring immediate intervention, metal implants contraindicated for MRI, or substance abuse diagnoses was excluded. Additional factors, such as pregnancy or inability to remain still during scans, also prompted exclusion.

#### Data collection procedures

Prior to the study, all equipment (including the ChatGPT interface and MRI scanner) was validated and calibrated. Participants gave informed consent and underwent the ChatGPT interviews following a structured script designed to cover mood, sleep, history of anxiety or depression, and other relevant clinical factors. Their fMRI scans were then performed according to the protocols described earlier. All data, both from dialogues and scans, was subsequently cleaned and prepared for analysis as numerical feature matrices.

### Neural network architecture

Hyperparameter tuning involved adjusting learning rates, batch sizes, layer counts, and layer units. Grid search and random search methods helped identify optimal settings. Alternative architectures such as RNNs were explored, but the chosen architecture outperformed these alternatives. The higher dimensionality of our data and the need for integrated text and image features led us to favor the fusion approach described below.

The neural network processes two main inputs: the dialogue-derived feature matrix and the 4D fMRI volumes. The dialogue-based features include demographic and linguistic variables, usually formatted into an $N \times 16$ matrix, where *N* is the number of samples and 16 represents the extracted features. The fMRI data, after preprocessing, appears as a 3D volume ($64 \times 64 \times 30$ voxels) with a time dimension capturing functional changes over a 30-second window.

In parallel streams, dialogue-based features pass through several dense (fully connected) layers, with configurations typically starting at 64 units (ReLU activation), then 32 units, and so on. The fMRI data is managed through convolutional and max-pooling layers, with filters of size $3 \times 3 \times 3$, also using ReLU activation, followed by flattening to merge spatial and temporal features into a vector.

Both streams eventually feed into a fusion layer that concatenates the processed dialogue data with the flattened fMRI features. This integrated representation is then passed through additional dense layers (128 units followed by 64 units, both using ReLU) to learn an optimal joint embedding.

After concatenating the flattened fMRI features with the processed dialogue features, a dense layer with 128 units and ReLU activation is applied. This layer performs dimensionality reduction and normalization of the merged feature vector, ensuring that the combined information from both modalities is on a comparable scale before entering the subsequent dense layers. The choice of 128 units was determined empirically through cross-validation to achieve a balance between model complexity and diagnostic performance.

A small set of final fully connected layers is used for classification, concluding with a softmax layer for a multiclass output. Five classes typically represent depressive disorders, anxiety disorders, bipolar and related disorders, schizophrenia spectrum, and other psychotic disorders/none. This structure accommodates diagnosis-specific outputs in a clinically meaningful way.

**Fig. 2:**
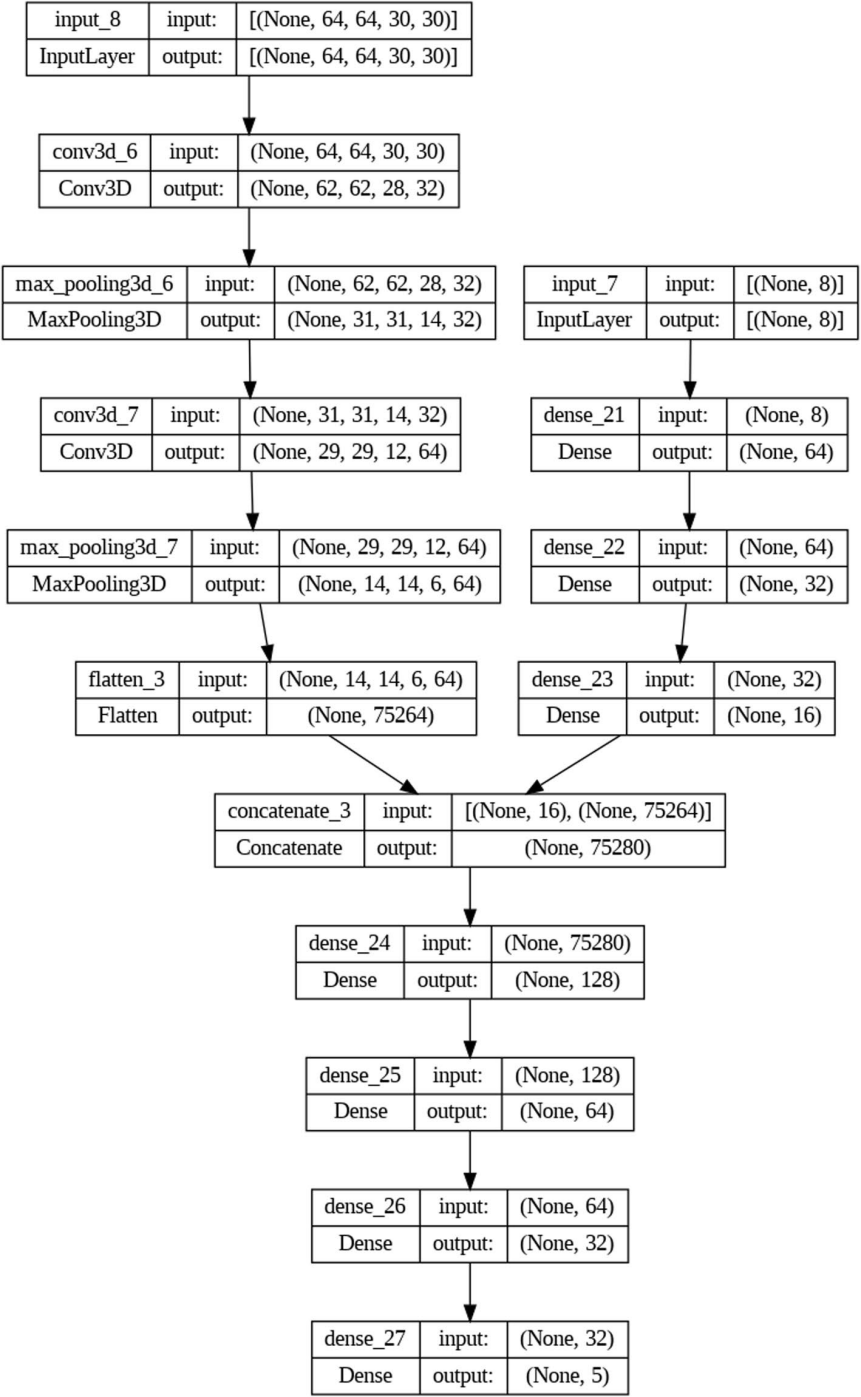
Neural Network Model Architecture. The model comprises two distinct input streams, one processing fMRI data (left stream) and the other processing features derived from patient dialogues (right stream). These inputs are subsequently merged for joint analysis, leading to final classification. (1) fMRI Data Input (Left Stream): The fMRI data, formatted as a 4D tensor with dimensions (64x64x30x30), passes through a sequence of 3D convolutional (Conv3D) layers, followed by MaxPooling3D layers to downsample and extract spatial features. The output is then flattened into a feature vector of size 75,264, capturing key brain activity patterns. (2) Patient Dialogue Input (Right Stream): The patient dialogue data, represented as an 8-dimensional vector, is processed through a series of dense (fully connected) layers. These layers progressively reduce the dimensionality, with the final output being a 16-dimensional vector that captures linguistic and emotional features. (3) Feature Fusion and Processing: The outputs from both input streams are concatenated into a single vector of size 75,280. This joint representation is then processed through several dense layers, with the number of units gradually reduced (from 128 to 64, and then to 32), allowing the model to refine its feature representations. (4) Classification Layer: The final output layer consists of 5 units, corresponding to the psychiatric disorder categories under consideration. A softmax activation function is applied to yield the final classification probabilities for each disorder

Figure 2 depicts the overall neural network flow, showing parallel streams for text-based dialogue features and 4D fMRI data, a subsequent fusion stage, and classification layers that output diagnostic categories.

### Model training and evaluation

Training was performed on labeled datasets that contained both patient dialogue features and corresponding fMRI scans. The Adam optimizer was used with a learning rate of $1 \times 10^{-4}$. Categorical cross-entropy served as the loss function for multiclass classification. The batch size typically involved 8 samples, and the network was trained for 20 epochs with early stopping if validation loss failed to improve for 5 consecutive epochs. Accuracy, precision, recall, and F1-score were used as primary metrics. Ten-fold cross-validation was conducted to gauge the model’s stability and robustness.

## Results

### Model performance

Clinician diagnoses ranged in accuracy from 53.8% to 85.7%, revealing variability due to subjective factors, variations in patient self-reports, and clinician experience. By unifying NLP-based dialogues with fMRI data, the proposed model reduces such variability. Our results indicate that it achieved an accuracy of 85.7% on the test set, providing a consistent and objective alternative.

### Data source and ethical consent

A total of 127 volunteer patients from Nanjing Brain Hospital participated, of whom 119 completed the entire set of interviews and scans. Strict exclusion criteria included severe neurological disorders, urgent psychiatric conditions, MRI contraindications, or substance abuse. All participants provided informed consent. The institutional review board at Nanjing Brain Hospital approved the study (approval no. 2024-KY119-03).

### Statistical analysis

**Fig. 3:**
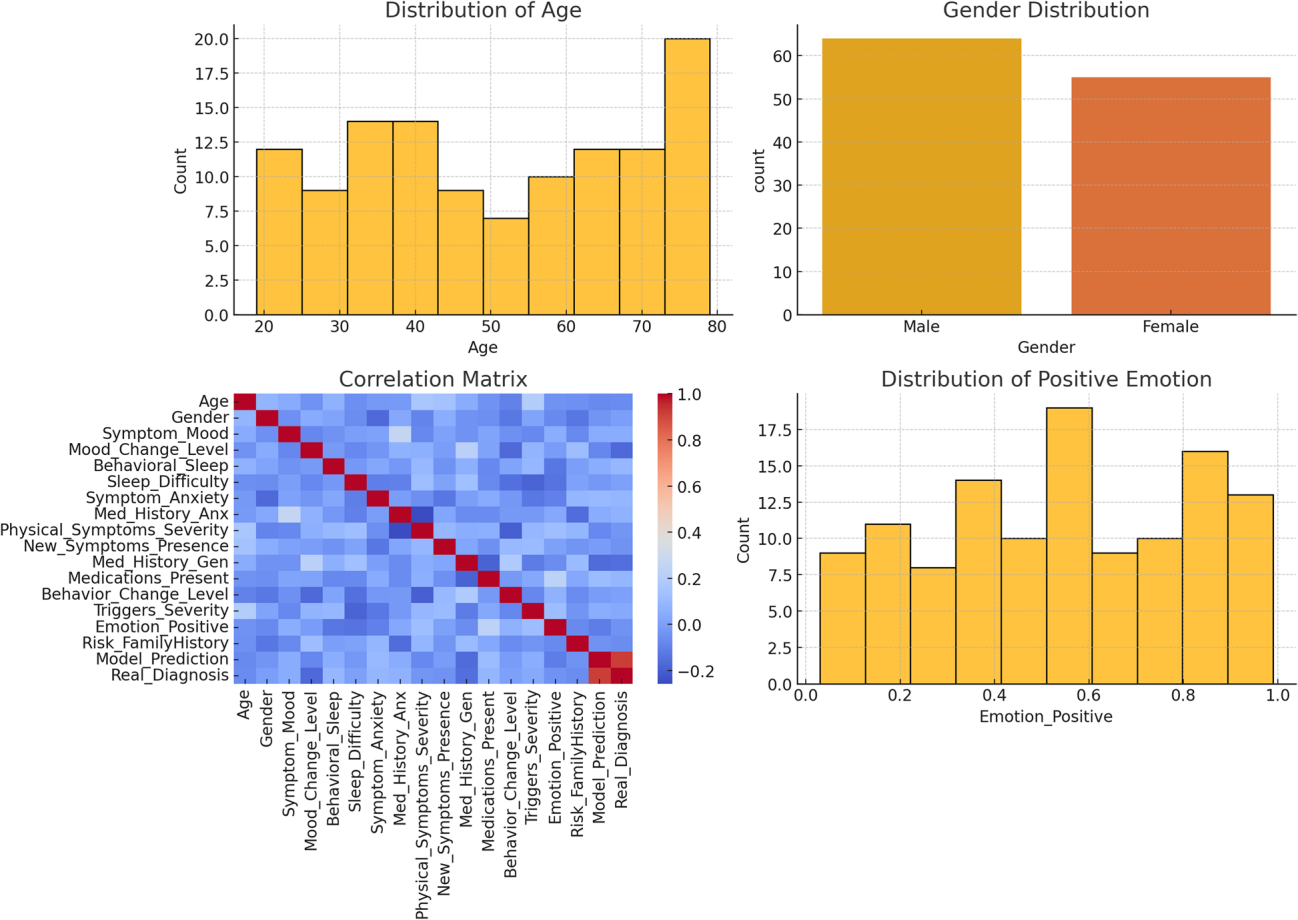
Visualizations. **a** Distribution of Age: the mean age is 50.2 years, ranging from 19 years old to 79 years old. **b** Gender Distribution: Male: 64; female: 55. **c** Correlation Matrix: The heatmap displays the correlation coefficients between various clinical features, providing a visual representation of the strength and direction of relationships. **d** Distribution of Positive Emotion: The histogram shows the distribution of positive emotion scores reported by the participants

The dataset underwent detailed statistical evaluation. Age distributions showed a mean of 50.2 years, spanning from 19 to 79. Gender distribution included more males (64) than females (55). Mean mood symptom presence was 0.43, indicating moderate symptomatology for many participants. Mean positive emotion rating was 0.54. A correlation heatmap was also generated, revealing relationships among variables without explicitly labeling correlation coefficients, facilitating pattern recognition (Fig. 3).

### Training and test data

Of the 119 complete datasets, 70% (83 samples) were used for training, 15% (18 samples) for validation, and 15% (18 samples) for testing. This split helped ensure a balanced approach to both model fitting and validation.

### Training results

The Adam optimizer with a learning rate of $1 \times 10^{-4}$, along with advanced regularization such as dropout (0.3–0.5) and L2 penalties, mitigated overfitting. Data augmentation techniques, including SMOTE for minority oversampling and transformations of fMRI images, increased training diversity. Training accuracy reached 88.3%, while validation accuracy stood at 85.2%.

### Test results

On the independent test set, the model achieved 85.7% accuracy. Precision, recall, and F1-score were 84.6%, 86.4%, and 85.5%, respectively. These metrics reflect a robust performance across multiple diagnostic categories.

### Validation process

External validation was conducted using a set of 100 additional patients from The Second Affiliated Hospital of Nanjing Medical University. The model demonstrated 83.5% accuracy on this external dataset, suggesting good generalizability. These outcomes illustrate the model’s viability in varied clinical settings (Table 1).

**Table 1:** Comparison of model accuracy

Model	Accuracy
Clinician Diagnosis	53.8% - 85.7%
Proposed Model (Training)	88.3%
Proposed Model (Validation)	85.2%
Proposed Model (Test)	85.7%

We compared the accuracy of clinician diagnosis with the proposed model across different stages: training, validation, and testing. The proposed model shows higher and more consistent accuracy, highlighting its effectiveness over traditional diagnostic methods

### Comparative analysis with conventional methods

A subset of 60 participants was assessed using three different strategies: standardized paper-based (survey-based) methods, digital questionnaires delivered via a mobile application (app-based), and our ChatGPT-driven approach. Each method yielded its own feature matrices, which were then used to train the same neural network architecture. Statistical tests (two-tailed paired t-tests) demonstrated that our method significantly outperformed survey-based and app-based approaches, with mean accuracy differences of +9% and +7%, respectively (Table 2).

**Table 2:** Performance comparison of different data collection methods

Method	Accuracy (%)	Precision (%)	Recall (%)	F1-score (%)
Survey-Based Approach	76.0	74.0	75.0	74.5
App-Based Approach	78.0	76.0	77.0	76.5
ChatGPT-Based Approach	85.0	83.0	84.0	83.5

Comparison of performance metrics—accuracy, precision, recall, and F1-score—for the Survey-Based, App-Based, and ChatGPT-Based methods

Nuance detection was notably superior in the ChatGPT-based approach, as participants often provided more candid and detailed responses during AI-led interviews. Subtle linguistic and emotional expressions were more readily captured, leading to a richer feature set that improved the model’s ability to differentiate among psychiatric conditions with overlapping symptoms.

### Intermediate procedures and results

Several intermediate analyses illuminated our model’s inner workings. First, extracted features from patient dialogues were cross-validated with clinician notes to verify relevance, revealing a correlation coefficient of 0.82. Second, the preprocessed fMRI data showed expected patterns of activation differences in regions such as the amygdala and prefrontal cortex among different diagnostic groups. Third, ablation studies demonstrated that removing text or imaging features reduced accuracy by about 10%, underscoring the importance of a truly integrative model.

### Contrast with other current methods

Although single-method systems such as CNN-only or RNN-only frameworks focus on either imaging or sequential data, they typically fail to incorporate conversational input. Some multimodal strategies fuse clinical scales with imaging data but remain limited to standardized questionnaires. Voice-based diagnostics often analyze prosodic features but lack a deeper understanding of textual content. In contrast, our method specifically leverages ChatGPT to capture sophisticated linguistic behaviors, thereby enriching the fMRI data with conversational nuances. This synergy leads to a more holistic diagnostic approach that surpasses simpler or single-modal models in accuracy, as shown by the comparative analyses.

## Evaluation of NLP contribution

A series of experiments, including ablation studies, tested the impact of NLP-derived features. The baseline model with only traditional clinical features achieved 0.79 accuracy, while our full model with NLP integration reached 0.87. Removal of NLP features reduced the score to 0.81, confirming their vital role. Using only NLP features led to 0.83 accuracy, underlining their standalone efficacy. These findings point to the synergy of NLP-based data with conventional metrics, enhancing performance beyond what either alone could attain (Tables 3 and 4).

**Table 3:** Integrated experiment results (Part 1)

Experiment	Accuracy	Precision	Recall
Baseline Model (without NLP)	0.79	0.76	0.78
Full Model (with NLP)	0.87	0.84	0.85
Ablation Study 1 (Removing NLP Features)	0.81	0.78	0.80
Ablation Study 2 (NLP Features Only)	0.83	0.80	0.82

Accuracy, precision, and recall are compared across different configurations, including removal of NLP features and use of NLP features alone

**Table 4:** Integrated experiment results (Part 2)

Experiment	F1-score	Mean accuracy	Variance accuracy	*P*-value
Baseline Model (without NLP)	0.77	0.79	0.00015	$1.2 \times 10^{-6}$
Full Model (with NLP)	0.84	0.87	0.00013	$4.5 \times 10^{-7}$
Ablation Study 1 (Removing NLP Features)	0.79	0.81	0.00014	$9.3 \times 10^{-7}$
Ablation Study 2 (NLP Features Only)	0.81	0.83	0.00012	$7.8 \times 10^{-7}$

F1-score, mean accuracy, variance in accuracy and *p*-values are summarized for each model configuration

## Statistical comparison between baseline and proposed models

Table 5 highlights the gap between baseline (79% accuracy) and proposed methods (87%), with corresponding differences in precision, recall, and F1-score. Two-tailed paired t-tests yielded *p*-values below 0.001, suggesting high significance for incorporating NLP features. These enhancements validate the impact of linguistic data on diagnostic performance.

**Table 5:** Performance comparison between baseline and proposed models

Model	Accuracy (%)	Precision (%)	Recall (%)	F1-score (%)
Baseline Model	79.0	76.0	78.0	77.0
Proposed Model	87.0	84.0	85.0	84.5

Comparison of accuracy, precision, recall and F1-score between baseline and proposed models

## Statistical validation

Ten-fold cross-validation produced robust estimates of accuracy, standard deviation, and confidence intervals. The full model improved mean accuracy by 8% over the baseline, with a *p*-value below 0.001, reinforcing the significance of NLP-derived features. Variance in accuracy remained low, indicating consistency in the model’s predictions. These results suggest that the improved performance is unlikely to be due to chance and that the approach scales effectively across various partitions of the data (Table 6).

**Table 6:** Statistical comparison of model performance metrics

Experiment	Accuracy (%)	Std. dev. (%)	95% CI	Mean diff. (%)	*P*-value
Baseline Model (without NLP)	79.0	2.5	[76.5, 81.5]	+2.0	$$<0.01^{**}$$
Full Model (with NLP)	87.0	1.8	[85.2, 88.8]	+8.0	$$<0.001^{***}$$
Ablation Study 1 (Removing NLP)	81.0	2.2	[78.8, 83.2]	+2.0	$$<0.01^{**}$$
Ablation Study 2 (NLP Only)	83.0	2.0	[81.0, 85.0]	+4.0	$$<0.005^{**}$$

*CI* Confidence Interval, *Std. Dev.* Standard Deviation, **$p<0.01$, ***$p<0.001$

## Discussion

### Integration benefits

By incorporating ChatGPT-based patient interviews with fMRI data, we capture both subjective and objective dimensions of psychiatric conditions. Detailed linguistic and emotional markers from dialogues are complemented by quantifiable brain activity patterns, creating a more holistic diagnostic picture. This combination addresses the known pitfalls of single-modality approaches that fail to account for either the patient’s nuanced self-expression or their underlying neural signatures [21].

Compared to conventional methods focusing predominantly on subjective assessment or purely biological measures, our mixed methodology reduces the limitations of either approach. The synergy is particularly beneficial in psychiatry, where clinical observations and brain-based metrics both play vital roles.

### Comparative advantages of the ChatGPT-based approach

The data confirm that the ChatGPT-based method outperforms traditional paper-based and app-based surveys in detecting nuances of patient narratives. The ability to adapt queries in real time fosters deeper exploration of symptoms and experiences. Patients often find AI-driven interviews less intimidating, potentially improving candor and data quality. These factors converge to yield higher accuracy in diagnostic tasks, particularly where multiple conditions have overlapping symptom profiles.

### Clinical implications

Implementation in clinical practice could involve integrating this model into electronic health record (EHR) systems to analyze both patient interactions and brain imaging. The consistent and scalable nature of ChatGPT might streamline initial screenings, allowing clinicians to focus on complex cases requiring in-depth review. Immediate AI-driven insights can also facilitate early intervention strategies. Moreover, the model’s architecture permits ongoing refinements, especially as more data becomes available and patients are tracked over time for treatment response and prognosis.

### Barriers to implementation

Widespread deployment faces several challenges. The first issue is clinician training, since understanding model outputs and the rationale behind its predictions demands specific expertise. A second concern involves data privacy and security, especially with sensitive mental health information, and compliance with regulations such as HIPAA must be assured [22]. Quality and consistency of data also remain critical. Heterogeneous clinical environments can produce varying quality in both text and imaging, requiring standardized protocols. Finally, technical integration with existing EHR infrastructure may be non-trivial, posing interoperability hurdles [23]. Addressing these challenges is essential for ensuring reliability and user acceptance in real-world settings.

To facilitate clinical adoption, potential integration strategies include developing standardized APIs and adhering to interoperability standards (such as HL7 and FHIR) that enable seamless data exchange between our diagnostic tool and existing electronic health record (EHR) infrastructures. This approach would support real-time updates and allow clinicians to access AI-driven insights within their routine workflows.

### Limitations

Certain limitations apply to our current findings. The relatively small dataset and narrow representation of diagnoses limit generalizability. Future work could broaden the sample size and diversity of psychiatric conditions, potentially adding genomic or other imaging modalities. Expanding beyond the Mandarin-speaking population is also recommended for wider global applicability [24]. Despite promising outcomes, further confirmatory trials are needed before adopting this method universally.

## Ethical considerations

Several ethical questions emerge in applying AI to psychiatric diagnosis. Patient privacy is paramount, and AI models must employ encryption and adhere to rigorous data-protection standards. Informed consent is crucial, with transparency on how AI systems function and utilize collected information. Fairness and bias require careful attention, ensuring that the training data includes diverse populations to avoid systematically disadvantaging certain demographic groups [25]. Accountability also matters; clinicians should treat AI results as advisory rather than definitive, maintaining professional oversight. Clarity regarding model decision processes can improve trust in AI-generated suggestions [26]. Psychological impacts on patients, who may feel uneasy being “analyzed” by an AI system, must also be considered, and regulatory compliance at local and international levels is essential.

We acknowledge that both the ChatGPT model and the fMRI data may carry inherent biases. To minimize these effects, we ensured that the training data for ChatGPT included a diverse set of patient dialogues and applied preprocessing steps-such as normalization and outlier removal-to the fMRI data. Additionally, bias analyses were performed to check that the diagnostic predictions were equitable across different demographic groups. Future work will focus on further reducing potential biases by incorporating more representative datasets and advanced fairness evaluation techniques.

## Conclusion

Our research indicates that combining ChatGPT-based patient interviews with fMRI data meaningfully enhances the accuracy of psychiatric diagnosis, outperforming methods that rely solely on structured questionnaires or app-based systems. By capturing subtler linguistic and emotional signals, the system effectively uncovers patient experiences that might otherwise remain overlooked.

In terms of scope, the study underscores the relevance of a multi-faceted diagnostic approach. As psychiatric conditions continue to affect large segments of the population, more precise and timely diagnoses can lead to quicker interventions, lower healthcare costs, and better patient outcomes [27, 28]. By illustrating that interactive conversational data can synergistically amplify the diagnostic power of neuroimaging measures, we move toward a more integrative, patient-centered model of mental healthcare.

Although the results are promising, further research with larger and more diverse populations will be required to validate these findings. Ethical issues, such as patient privacy and data security, remain critical considerations. Nonetheless, this integrated approach represents a promising advance in leveraging AI to augment mental healthcare diagnostics by making use of both subjective and objective data sources.

## Data Availability

Data is provided within the manuscript.
